# Toward a three-dimensional view of protein networks between species

**DOI:** 10.3389/fmicb.2012.00428

**Published:** 2012-12-21

**Authors:** Eric A. Franzosa, Sara Garamszegi, Yu Xia

**Affiliations:** ^1^Bioinformatics Program, Boston UniversityBoston, MA, USA; ^2^Department of Chemistry, Boston UniversityBoston, MA, USA; ^3^Department of Biomedical Engineering, Boston UniversityBoston, MA, USA; ^4^Center for Cancer Systems Biology and Department of Cancer Biology, Dana-Farber Cancer InstituteBoston, MA, USA

**Keywords:** structural systems biology, protein–protein interaction, host–pathogen interaction, bioinformatics and computational biology, network biology

## Abstract

General principles governing biomolecular interactions between species are expected to differ significantly from known principles governing the interactions within species, yet these principles remain poorly understood at the systems level. A key reason for this knowledge gap is the lack of a detailed three-dimensional (3D), atomistic view of biomolecular interaction networks between species. Recent progress in structural biology, systems biology, and computational biology has enabled accurate and large-scale construction of 3D structural models of nodes and edges for protein–protein interaction networks within and between species. The resulting within- and between-species structural interaction networks have provided new biophysical, functional, and evolutionary insights into species interactions and infectious disease. Here, we review the nascent field of between-species structural systems biology, focusing on interactions between host and pathogens such as viruses.

## INTRODUCTION

Protein–protein interactions (PPIs) can be divided into two fundamentally different classes. The first class of PPIs involves interactions between two proteins encoded within the genome of a single species, where the two proteins cooperate with each other to achieve cellular function in a coordinated fashion. The second class of PPIs involves interactions between two proteins from different species, for example between host proteins and microbial proteins, or between proteins from two different microbial species. These between-species PPIs play key roles in host–microbe and microbe–microbe interactions. Unlike the cooperative PPIs within the host, the interactions between host and microbes are driven by a wide spectrum of co-evolutionary mechanisms, ranging from parasitic to mutualistic (Dethlefsen et al., 2007). General principles of the PPI networks between microbes and their host may differ significantly from known principles governing the cooperative PPI network encoded within the host, yet these principles are not well understood. Here, we review recent progress toward constructing a high-resolution, three-dimensional (3D) structural view of host–pathogen and within-host PPI networks. The resulting host–pathogen and within-host structural interaction networks enable the discovery of new principles of host–pathogen interactions that are otherwise hidden in the binary PPI network. This review focuses on high-throughput mapping and large-scale analysis of host–pathogen PPI networks, which reveal global trends and patterns in host–pathogen interactions that are minimally confounded by investigator biases.

## HOST–PATHOGEN PROTEIN–PROTEIN INTERACTION NETWORKS

The first step toward building host–pathogen structural interaction networks is to map the networks of physical interactions between host proteins and pathogen proteins. Host–pathogen PPIs have traditionally been studied one at a time. Recently, systems biology approaches have been applied to host–pathogen interaction research. Significant progress has been made in genome-wide mapping of host–pathogen PPI networks (“interactomes”) for many pathogens, especially viruses. Using high-throughput methods such as the yeast two-hybrid system (Fields and Song, 1989) and affinity purification followed by mass spectrometry identification (Rigaut et al., 1999), experimental host–pathogen interactome maps now exist for many viruses (von Schwedler et al., 2003; Uetz et al., 2006; Calderwood et al., 2007; de Chassey et al., 2008; Shapira et al., 2009; Zhang et al., 2009; Khadka et al., 2011; Jager et al., 2012; Pichlmair et al., 2012; Rozenblatt-Rosen et al., 2012). Since viruses are obligate intracellular parasites with small genomes, many, but not all, physical interactions between viral proteins and host proteins have functional importance. Thus, it is essential to complement physical interactome mapping with functional assays that identify host proteins whose perturbation significantly affects viral infection and replication (Brass et al., 2008; Konig et al., 2008, 2010; Krishnan et al., 2008; Karlas et al., 2010). In addition to host–virus interactome maps, limited host–pathogen interactome data exist for bacterial and eukaryotic pathogens (Dyer et al., 2008, 2010; Mukhtar et al., 2011; Schleker et al., 2012). Since most proteins in bacterial and eukaryotic pathogens do not directly interact with host proteins, a key challenge is to identify pathogen effector proteins that act directly on the host cell to enable infection (Tobe et al., 2006).

Experimental host–pathogen interactome datasets are expected to continue to expand in the near future. The many thousands of experimentally detected host–pathogen PPIs are collected in databases such as VirusMINT (Chatr-aryamontri et al., 2009), VirHostNet (Navratil et al., 2009), IntAct (Aranda et al., 2010), PIG (Driscoll et al., 2009), and NCBI HIV-1 protein interaction database (Fu et al., 2009). These databases typically rely heavily on manual curation to maintain standards of quality, and there is a great need to complement manual curation with automated literature mining of host–pathogen PPIs (Thieu et al., 2012).

Because of the challenges associated with experimental determination of host–pathogen PPIs, it is desirable to develop computational methods to predict host–pathogen PPIs. Prediction of host–pathogen PPIs is usually based on sequence homology with known PPIs (Uetz et al., 2006; Davis et al., 2007; Doolittle and Gomez, 2011; Wuchty, 2011), the presence of known or predicted interacting domain pairs (Dyer et al., 2007), as well as the presence of other predictive sequence and functional features (Tastan et al., 2009; Qi et al., 2010; Dyer et al., 2011). Computational predictions of host–pathogens PPIs are most effective as a means to prioritize subsequent experimental validations, which are often time-consuming (Uetz et al., 2006). Other areas where computational methods play an increasingly important role include genomic data integration of diverse host–pathogen physical, genetic, and functional interactions (Shapira et al., 2009; Konig et al., 2010; Rozenblatt-Rosen et al., 2012), and network-based prediction of host proteins important in host–pathogen interaction (Navratil et al., 2010; Murali et al., 2011).

Experimental host–pathogen PPI networks are useful in many ways. They not only help generate hypotheses regarding the function of specific pathogen proteins and the biology of specific pathogens, but also provide insights into principles governing host–pathogen interactions at the systems level. Global analyses of host–pathogen PPI networks have revealed that viruses and other microbial pathogens tend to interact with host proteins that are hubs (i.e., proteins with many interaction partners in the host network) and bottlenecks (i.e., proteins whose removal would disrupt many shortest paths in the host network; Calderwood et al., 2007; de Chassey et al., 2008; Dyer et al., 2008; Wuchty et al., 2010; Pichlmair et al., 2012). Host proteins that interact with pathogens tend to be conserved among closely related species (Jager et al., 2012; Pichlmair et al., 2012), although many of them are also under positive selection (Bozek and Lengauer, 2010). Host proteins that interact with pathogens tend to form densely connected network modules by clustering into biological pathways and physical complexes (Dyer et al., 2008; Bushman et al., 2009; MacPherson et al., 2010). In addition, host–pathogen PPI networks are enriched for certain network motifs (e.g., mutual inhibition; van Dijk et al., 2010). Furthermore, pathogens tend to target host proteins involved in common biological processes essential to pathogen infection and replication in general, such as host defense and immune response (Dyer et al., 2008; Pichlmair et al., 2012), often through convergent evolution (Mukhtar et al., 2011). At the same time, different classes of pathogens (e.g., DNA viruses versus RNA viruses, or viruses versus bacteria) also target distinct host pathways due to class-specific differences in infection and replication mechanisms (Durmus Tekir et al., 2012; Pichlmair et al., 2012). Finally, host proteins targeted by pathogens tend to be in network proximity to other proteins implicated in diseases associated with pathogen infections (Navratil et al., 2011; Gulbahce et al., 2012). It is clear that much can be learned by taking a global and network perspective on host–pathogen interactions.

## HOST–PATHOGEN STRUCTURAL INTERACTION NETWORKS

**FIGURE 1:**
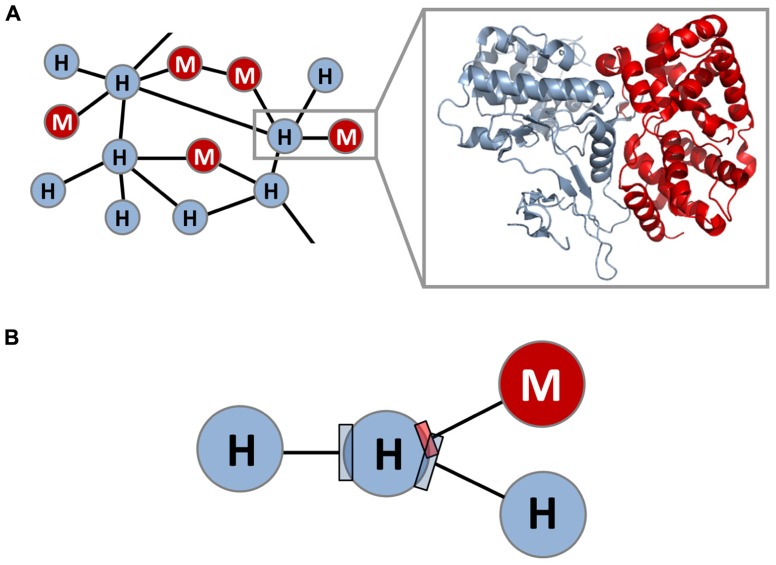
**Structural interaction network between species**. **(A)** Shown is a high-resolution, 3D structural view of the PPI network between host and microbial pathogens, where each within-host and host–microbe protein–protein interaction (PPI) edge is associated with an accurate 3D structural model; one such interaction (gray box) and its structural model are highlighted. Interactions can be within human or within microbe (within-species interactions), or between human and microbe (between-species interactions). **(B)** The resulting host–microbe structural interaction network reveals high-resolution geometrical relationships between exogenous interfaces (between-species interfaces) and endogenous interfaces (within-species interfaces) that are otherwise hidden in the binary PPI network. In this example, a microbial protein is seen to bind to a target protein in the host at the same site as another host protein, albeit using a smaller interface.

The mapping of host–pathogen PPI networks lays the foundation for and constitutes the first step toward constructing host–pathogen structural interaction networks. Despite experimental and computational advances in the global analysis of host–pathogen PPI networks, the utility of PPI networks is ultimately limited by their low-resolution nature (i.e., proteins represented as nodes and PPIs represented as edges). A high-resolution view of the host–pathogen PPI network can be achieved by building accurate 3D structural models for nodes and edges in the network (**Figure 1A**). Is it feasible to construct such a host–pathogen structural interaction network in a global and accurate way? And if so, does this 3D structural view provide new insights into host–pathogen interactions that are not apparent in the binary PPI network?

Although the 3D structure of proteins and PPIs can in principle be predicted from sequence without resorting to homology [using template-free structure prediction (Moult, 2005) and macromolecular docking (Gray, 2006)], in practice homology modeling remains the most successful and reliable 3D structure prediction method on a genomic scale for both proteins and PPIs (Marti-Renom et al., 2000; Russell et al., 2004). To build a homology model for a query protein or a query pair of interacting proteins, the query protein or protein pair is searched against a template library consisting of proteins or PPIs of known 3D structure deposited in the Protein Data Bank (PDB; Berman et al., 2000). The most significantly matched 3D template is then used to construct a homology model for the query protein or PPI. Despite the obvious limitations that good homology models cannot be built for proteins with entirely new folds or PPIs with entirely new modes of interaction, and that the conformation of proteins and PPIs is not always conserved during evolution, homology modeling has been highly successful in practice, thanks to major advances in structural biology and computational biology. Proteins and PPIs are composed of a limited number of domains and domain–domain interactions (Chothia, 1992; Aloy and Russell, 2004), and certain domains and domain–domain interactions are significantly overrepresented in proteomes and interactomes (Qian et al., 2001). Thus, homology models for many proteins and PPIs can be built based on a relatively small number of representative domains and domain–domain interactions of known 3D structure, stored in databases such as SUPERFAMILY (Madera et al., 2004), iPfam (Finn et al., 2005), and 3did (Stein et al., 2005). Indeed, it is estimated that ~60% of all query proteins share significant sequence similarity with at least one template protein of known 3D structure (Madera et al., 2004). For the vast majority of these cases, the query protein shares significant structural similarity with the template protein, an accurate sequence alignment can be constructed, and an accurate homology model (~3 Å RMSD) can be built for at least a part of the query protein (typically a domain; Marti-Renom et al., 2000; Dalton and Jackson, 2007). Compared to homology modeling of single proteins, the coverage of accurate homology models for within-species PPIs is smaller but still considerable (~20%; Kim et al., 2006). Indeed, it was recently argued that 3D templates exist for most known within-species PPIs, provided that good homology models can be built for the protein components (Kundrotas et al., 2012). The coverage of accurate template-based models for PPIs can be further improved by identifying additional 3D templates that are structurally similar to the query proteins in the absence of sequence similarity (Zhang et al., 2012).

Homology modeling has been successfully used to construct within-species structural interaction networks, where 3D structural models are built for known within-species PPIs (Aloy et al., 2004; Kim et al., 2006). Despite the caveat that 3D homology models are biased toward soluble, stable, and structurally well-ordered proteins and PPIs, structural interaction networks can be viewed as high-quality subsets of binary PPI networks with much higher spatial resolution. Computational analyses of the within-species structural interaction networks have provided significant insights into a wide range of topics including biophysics, evolution, disease biology, and drug design (Kim et al., 2006, 2008; Franzosa and Xia, 2008, 2009; Kar et al., 2009; Xie et al., 2011; Wang et al., 2012). Such structural systems biology approaches are highly valuable as a unifying framework that integrates molecular biophysics with cell systems biology.

Most recently, structural systems biology was applied to between-species interactions, and an integrated map of human–virus and within-human structural interaction networks was constructed (Franzosa and Xia, 2011). The structural interaction networks consist of 53 human-virus PPIs and >3,000 human–human PPIs in the form of either experimental 3D structures or homology models. Here, instead of predicting new host–pathogen PPIs (Davis et al., 2007), homology modeling is used to annotate known host–pathogen PPIs with 3D structural information, thus providing a structural map of the binary PPI network in much higher spatial resolution. For example, the binary PPI network indicates that the human CDK6 protein interacts with both human proteins and the cyclin D homolog protein from herpesvirus. The structural interaction network further reveals that these interactions largely occur at two distinct, non-overlapping interfaces on the human CDK6 protein: one interface mediating the interactions with the viral protein as well as the human cyclin D protein, and a second interface mediating the interactions with various human CDK inhibitor proteins (Russo et al., 1998; Pratt et al., 2006). Such a high-resolution map enables the detailed analysis of the geometrical properties and relationships of human–virus PPI interfaces (exogenous interfaces) and human–human PPI interfaces (endogenous interfaces) that is otherwise inaccessible in the binary PPI network (**Figure 1B**; Franzosa and Xia, 2011). For example, although binary PPI network analysis revealed that viral proteins tend to interact with host protein hubs participating in many endogenous interactions, the precise spatial relationships among these exogenous and endogenous interactions are not known. On the other hand, structural interaction analysis further revealed that exogenous interfaces, although smaller in size, tend to overlap significantly with and mimic endogenous interfaces, often in the absence of sequence or structural similarity. In addition, the endogenous interfaces that are mimicked by viral proteins tend to participate in multiple endogenous interactions which are transient and regulatory in nature. A case in point is the interaction between the UL36 protein from the HSV-1 virus and the human ubiquitin protein, an important regulator of protein function and cell behavior (Schlieker et al., 2007). The endogenous interface of the human ubiquitin protein mimicked by the virus mediates as many as 30 interactions with other human proteins. On average an endogenous interface mimicked by virus mediates more than three interactions with other human proteins in the structural interaction network, whereas a generic endogenous interface only mediates ~1.5 interactions with other human proteins. These observations demonstrate that viral proteins tend to mimic and hijack high-level regulatory components of the host cellular circuitry, by efficiently binding to existing endogenous interfaces rather than creating entirely new interfaces. Furthermore, endogenous interfaces mimicked by viral proteins tend to evolve more quickly than other endogenous interfaces, suggesting an evolutionary “arms race” between host and pathogen. Overall, 3D structural analysis revealed, in a systematic and statistically rigorous way, distinct principles governing antagonism versus cooperation in host–pathogen and within-host PPI networks (Franzosa and Xia, 2011).

Protein–protein interactions can be divided into two classes: the first class involves PPIs mediated by interactions between two globular domains, and the second class involves PPIs mediated by short linear motifs interacting with globular domains. Both classes are important mediators of host–pathogen interactions (Davey et al., 2011; Franzosa and Xia, 2011). A recent survey revealed extensive mimicry of host short linear motifs by viruses (Davey et al., 2011). Viral mimicry of host linear motifs was found for 52 of the ~150 motif classes in the Eukaryotic Linear Motif (ELM) database (Gould et al., 2010), 13 of which have solved 3D structures involving viral motifs in complex with their host targets. For example, there are many cases of viral proteins targeting the SH3, SH2, or PDZ domains of host proteins using mimicked motifs. These observations are in agreement with the requirements for viral proteins to extensively hijack and manipulate diverse host proteins and pathways, despite the severe spatial constraints imposed by their small genomes (Davey et al., 2011). These motifs tend to cluster into hotspots in the viral genome (Sarmady et al., 2011), and they may be important determinants of virulence (Yang, 2012). While motifs play an important role in the biology of viruses and viruses use motifs extensively, it is not known if viruses use motifs more often than the host (Davey et al., 2011). These findings collectively highlight the feasibility and importance of structural systems biology in host–pathogen interaction research.

## CONCLUSION

Despite being a relatively new field, between-species structural systems biology has already provided major insights into species interactions and infectious disease. We expect to see rapid growth in between-species structural systems biology over the next few years on the following fronts. First, host–pathogen physical, genetic, and functional interaction datasets will continue to accumulate for more pathogens, and with higher coverage and accuracy. The impact of these interactions on host and pathogen physiology will continue to be systematically evaluated. In addition to interaction data, small-scale experiments and large-scale technologies such as genome-wide association studies (Khor and Hibberd, 2012) have generated large amounts of data describing mutations that affect host–pathogen interaction and pathogenicity. A key computational challenge is the development of unified, predictive models of how host and pathogens interact through integration of these datasets. Second, the success of homology modeling depends critically on the availability of 3D structural templates for representative proteins and PPIs solved by experimental structural biologists. The power of homology modeling is especially limited for fast-evolving pathogens such as viruses, where experimental structural biology plays a central role. It is encouraging that the number of 3D structures of human–virus PPIs have doubled in the past 5 years (Franzosa and Xia, 2012), and we expect a significant expansion in the number of 3D structures for host–pathogen PPIs in the next few years. Structural genomics has been highly successful by focusing primarily on structure determination of single proteins (Chandonia and Brenner, 2006). It will be fascinating to investigate if high-throughput structural biology can be applied to within- and between-species PPIs as well. Finally, new methods will be developed to integrate interaction datasets with 3D structure datasets. Computational analysis of the resulting structural interaction networks will uncover new system-level insights into host–pathogen interactions.

## Conflict of Interest Statement

The authors declare that the research was conducted in the absence of any commercial or financial relationships that could be construed as a potential conflict of interest.
